# Mental health and preventive behaviour of pregnant women in China during the early phase of the COVID-19 period

**DOI:** 10.1186/s40249-021-00825-4

**Published:** 2021-03-24

**Authors:** Qian Wang, Phoenix K. H. Mo, Bo Song, Jiang-Li Di, Feng-Rong Zhou, Juan Zhao, Ying-Lan Wu, Hong Tian, Li-Qian Qiu, Jianhong Xia, Lan Wang, Fen Li, Lin-Hong Wang

**Affiliations:** 1 National Center for Women and Children’s Health, Chinese Center for Disease Control and Prevention, Beijing, China; 2 Center for Health Behaviours Research, School of Public Health and Primary Care, The Chinese University of Hong Kong, Hong Kong, China; 3 Shandong Province Hospital for Women and Children’s Health, Jinan, China; 4 Beijing Obstetrics and Gynecology Hospital, Capital Medical University, Beijing, China; 5 Hunan Provincial Maternal and Child Health Care Hospital, Changsha, China; 6 Women and Children’s Health Care Hospital of Liaoning Province, Shenyang, China; 7 School of Medicine, Women’s Hospital, Zhejiang University, Hangzhou, China; 8 Guangdong Women and Children Hospital, Guangzhou, China; 9 Chong Qing Health Center for Women and Children, Chong Qing, China; 10 First Affiliated Hospital of Medical College of Xi’an Jiaotong University, Xi’an, China; 11 National Center for Chronic and Noncommunicable Disease Control and Prevention, Chinese Center for Disease Control and Prevention, 13# Rd Nanwei, Xicheng, Beijing, China

**Keywords:** COVID-19, Personal preventive behaviour, Depression, Anxiety, Mental health, Pregnant women, China

## Abstract

**Background:**

The COVID-19 has caused significant toll over the globe. Pregnant women are at risk of infection. The present study examined the frequency of washing hands with soap and wearing face mask when going out, prevalence of depression and anxiety, and identified their associated factors among pregnant women during the early phase of COVID-19 outbreak in China.

**Methods:**

A cross-sectional online survey was conducted between 24 February and 3 March 2020. A total of 15 428 pregnant women who were using maternal health care services in China completed a questionnaire which assessed their socio-demographic and pregnancy-related characteristics, contextual, cognitive and social factors related to COVID-19, frequency of washing hands and wearing face masks, and depression and anxiety. Logistics regression analyses were performed to identify the associated factors of preventive behaviours and mental health.

**Results:**

The prevalence of probable anxiety and depression was 28.2% and 43.6% respectively. 19.8% reported always wearing face mask when going out, and 19.1% reported washing hands with soap for more than 10 times per day. Results from logistic regression analyses showed that older age was associated with lower levels of depression and anxiety (*OR* = 0.42–0.67) and higher frequency of washing hands (*OR* = 1.57–3.40). Higher level of education level was associated with probable depression (*OR* = 1.31–1.45) and higher frequency of wearing face mask (*OR* = 1.50–1.57). After adjusting for significant socio-demographic and pregnancy-related factors, place of residence being locked down (a*OR* = 1.10–1.11), being quarantined (a*OR* = 1.42–1.57), personally knowing someone being infected with COVID-19 (a*OR* = 1.80–1.92), perception that COVID-19 would pose long term physical harm to human (a*OR* = 1.25–1.28) were associated with higher levels of depression and anxiety, while the perception that the disease will be under control in the coming month was associated with lower levels of depression and anxiety (a*OR* = 0.59–0.63) and lower tendency of always wearing face mask (a*OR* = 0.85). Social support was associated with lower levels of depression and anxiety (a*OR* = 0.86–0,87) and higher frequency of washing hands (a*OR* = 1.06).

**Conclusions:**

The mental health and preventive behaviours of pregnant women during COVID-19 outbreak was associated with a range of socio-demographic, pregnancy-related, contextual, cognitive and social factors. Interventions to mitigate their mental health problems and to promote preventive behaviours are highly warranted.

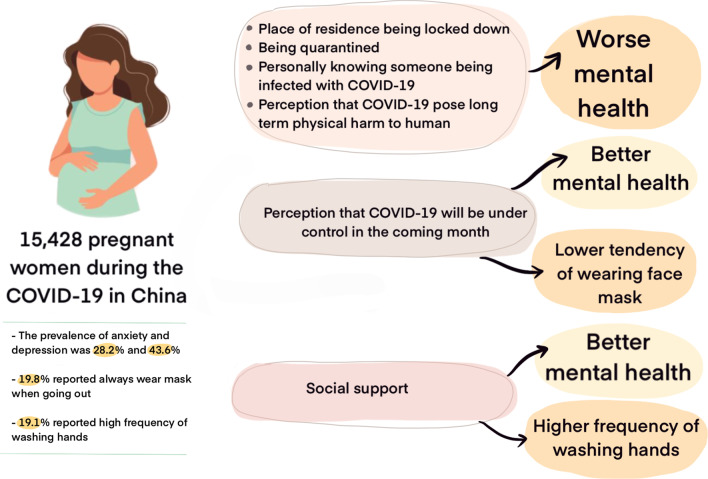

**Supplementary Information:**

The online version contains supplementary material available at 10.1186/s40249-021-00825-4.

## Background

The coronavirus disease 2019 (COVID-19) is a recent emerging infectious disease that has caused significant morbidity and severe threat to public health across the globe. As of 28 December 2020, 96 417 COVID-19 confirmed cases were reported and 4778 COVID-19 related deaths occurred in China [1]. More than 79 million (79 673 754) of confirmed cases of COVID-19 have been reported worldwide, with 1 761 381 deaths [1]. Pregnant women are particularly affected by an epidemic outbreak as any infection or morbidity would affect both the mother and the child. Studies in respiratory epidemics, such as severe acute respiratory syndrome (SARS) and middle east respiratory syndrome (MERS) have found that pregnancy was associated with higher incidence of adverse maternal and neonatal complications [2, 3]. It would be essential to understand the behavioural and mental responses to the COVID-19 outbreak of pregnant women, so that tailored interventions can be developed to mitigate the negative impact of the outbreak in this population.

In non-pandemic time, one in seven pregnant women experienced depression and anxiety during the perinatal period [4]. The limited studies on pregnant women during an epidemic outbreak have suggested that pregnant women tended to show high level of worry about themselves and the child’s condition, and poor mental health [5, 6]. In the context of COVID-19, one recent study among 900 pregnant women in Canada found that compared to the pre-COVID-19 period, pregnant women showed elevated prevalence of depression (from 15 to 41%) and anxiety (from 29 to 72%) during the COVID-19 pandemic [7].

The behaviour of individual also plays an important role in the control of infectious diseases. Behaviours such as maintaining hand hygiene and wearing face masks have been recommended as prevention strategies against COVID-19 [8, 9]. On 5 June 2020, the World Health Organization (WHO) released its updated advice that the general public should wear a face mask where there is known or suspected widespread transmission and where physical distancing is not possible. In the context of COVID-19, one study among general public in Wuhan and Shanghai, China in the early phase of the pandemic reported that frequency of always wearing a face mask surged drastically from 9.2–12.2% at usual days, to 76.1–85.5% in the past week, while the frequency of always washing hands immediately after returning home increased from 42.4 to 56.3% at usual days, to 74.3–79.3% in the past week [10]. It would be important to understand the different strategies that pregnant women adapt to mitigate their risk of contracting the infection.

It is important to identify the factors associated with preventive behaviours and mental health among pregnant women. Research among pregnant women has shown that younger age, lower socioeconomic status and more socioeconomic impact of the pandemic were associated with mental health problems [6, 11, 12]. Pregnancy complications [12] was identified as significant risk factor, while social support was a significant protective factor for mental health and health behaviours among pregnant women [12, 13].

Perceptions on the disease, such as perceived risk and extent of harm of the disease, were significant factors to mental health and health behaviours. Studies among the Chinese public during the COVID-19 outbreak have found that higher perceived risk and severity of contracting the virus, higher perceived relative transmissibility and harm to body compared to SARS, and lower perceived controllability were associated with negative mental outcomes and precautionary behaviours [10, 14]. Contextual factor such as being quarantined [15, 16] was also significantly related to poor health outcomes during a disease outbreak.

### The present study

The present study assessed the prevalence of personal preventive behaviour (i.e. frequency of washing hands with soap and wearing face mask) and mental health (i.e. depression and anxiety) among pregnant women during the COVID-19 outbreak in China. Factors associated with the behavioural and mental health outcomes, including socio-demographic variables (i.e. age, level of education), pregnancy-related (i.e. parity, gestational age and pregnancy complications), contextual (i.e. being quarantined, place of residence being locked down, personally knowing someone infected with COVID-19), cognitive (i.e. perception of risk and level of harm of COVID-19) and social factors (i.e. social support obtained during the outbreak) were also explored.

## Methods

### Participants

An online cross-sectional questionnaire survey was conducted among pregnant women from 24 February to 3 March 2020. Inclusion criteria were: (1) female, (2) aged 18 years or older, (3) Chinese speaking, (4) currently pregnant, and (5) using maternal health care services of Maternal and Child Health Hospitals of the Chinese Preventive Medicine Association from all provincial-level administrative regions in China. Those who planned to terminate their pregnancy were excluded from the study. Informed consents were obtained from all participants. Ethical approval was obtained from the Survey and Behavioral Research Ethics Committee of the Chinese University of Hong Kong.

### Procedure

Antenatal care providers of each institution first identified eligible participants from their database and invited eligible participants through Wechat. A Quick response (QR) code and a direct link to the online questionnaire was included in the invitation message. By scanning the QR code or directly clicking the link on the mobile phone or tablet devices, interested participants could enter the online questionnaire. All pertinent information about the study (e.g. purpose, procedures, estimated risk) was presented and explained in the title page. Confidentiality and anonymity were also assured. Informed consent was obtained by asking the participants to click the “I agree” button before they started the online survey. The entire survey took 15 to 20 min to complete. Participants received no incentive for completing the study. Only those who have provided complete information was retained in the survey database. A total of 15 428 responses were obtained.

### Sample size calculation

Based on the literature, it is assumed that around 40% of pregnant women would have probable depression during the COVID-19 period [7]. The sample size of 15 428 allows us to estimate the prevalence of anxiety with a margin of error of ± 0.7% at 95% confidence. The target sample size of 15 428 allows us to detect smallest odds ratio of 1.08 (80% power and 5% level of significance; GPower version 3.13 [17]).

### Measures

Details of the measures used in the present study are presented in Additional file 1. Socio-demographic characteristics including age, education level, place of residence, and pregnancy-related characteristics including parity, gestational age, and whether they had any pregnancy-related complications were collected. Participants were also asked to report whether their place of residence was locked down, whether they have been quarantined, and whether someone they personally knew were infected with COVID-19.

*Cognitive factors*. Participants were asked to rate the likelihood that the COVID-19 would be under control in the coming month, and if the COVID-19 would pose long term physical harm to human.

*Social factor*. Participants were asked to rate the level of social support they have obtained during the COVID-19 outbreak period.

*Personal preventive behaviors*. Participants were asked to report their frequency of wearing a face mask when going out, and their frequency of washing hands with soap everyday.

*Mental health*. Depression was measured by the 9-item Chinese version of Patient Health Questionnaire-9 (PHQ-9) [18]. Anxiety was assessed by the 7-item Chinese version of General Anxiety Disorder scale (GAD-7) [19].

### Data analysis

Descriptive statistics on participants’ socio-demographic and pregnancy-related characteristics, contextual factors, cognitive factors and social factor were presented. The prevalence of response variables among participants from different regions (i.e. Northern, Eastern, Southern, Central, Northwest China) was also presented. There were four response variables in the present study: probable depression (PHQ-9 > 5), probable anxiety (GAD-7 > 5), always wearing face mask, and washing hands with soap for more 11 times or more per day. Spearman’s correlation coefficient between mental health (i.e. depression and anxiety) and preventive behaviors (i.e. frequency of wearing face mask and washing hands with soap) were first examined. Univariate logistic regressions were conducted to examine the association between all the independent variables and the response variables. Multiple logistic regressions were then fit for each of the individual contextual factors, cognitive factors, and social factor on each response variable, adjusted for socio-demographic and pregnancy-related variables that were statistically significant in the simple logistic regressions as control variables. Data analysis was performed using SPSS version 16.0 (IBM SPSS Statistics, USA) with *P* value of < 0.05 being considered as statistically significant.

## Results

### Descriptive statistics

Of all respondents, 59.9% were 30 years old or below; 35.4% had attained university education or above; most of them came from Shandong (25.8%) and Zhejiang (23.7%) provinces. More than half of them (56.5%) were nulliparous, and half of them (49.8%) were in the 3rd trimester.

Over 60% (60.9%) reported that their place of residence has been locked down, and 3.8% have been quarantined during the COVID-19 epidemic. A vast majority of them (91.9%) believed that the COVID-19 would be under control in the coming month, and 57.8% believed that the COVID-19 would pose long term physical harm to human. Their mean score of social support obtained during the epidemic was 8.6/10 [Standard deviation (*SD*) = 2.03] (Table 1).

**Table 1:** Characteristics of the participants (*N* = 15 428)

Characteristics	*N* (%)
*Socio-demographic characteristics*	
Age, years	
19 or below	178 (1.2%)
20 to 25	2674 (17.3%)
26 to 30	6388 (41.4%)
31 to 35	4628 (30.0%)
36 to 40	1325 (8.6%)
41 or above	235 (1.5%)
Education level	
Primary or below	275 (1.8%)
Junior secondary	2556 (16.6%)
Senior secondary	2959 (19.2%)
Matriculation	4171 (27.0%)
Undergraduate	4553 (29.5%)
Postgraduate or above	914 (5.9%)
Place of residence	
Northern China	
Liaoning	680 (4.4%)
Beijing	1845 (12.0%)
Eastern China	
Shandong	3975 (25.8%)
Zhejiang	3657 (23.7%)
Southern China	
Guangdong	806 (5.2%)
Shenzhen	870 (5.6%)
Guangxi	562 (3.6%)
Chongqing	635 (4.1%)
Central China	
Henan	286 (1.9%)
Hunan	1259 (8.2%)
Northwest China	
Shaanxi	525 (3.4%)
Qinghai	327 (2.1%)
*Pregnancy-related characteristics*	
Parity	
Nulliparous	8719 (56.5%)
Primiparous	5976 (38.7%)
Multiparous	733 (4.8%)
Gestational age	M = 25.79, *SD* = 9.40
1st trimester (12 weeks or below)	1467 (9.5%)
2nd trimester (13 to 26 weeks)	6280 (40.7%)
3rd trimester (27 weeks or above)	7681 (49.8%)
Pregnancy-related complications	
No	14 040 (91.0%)
Yes	1388 (9.0%)
*Contextual factors*	
Place of residence being locked down	
No	6035 (39.1%)
Yes	9393 (60.9%)
Being quarantined	
No	14 849 (96.2%)
Yes	579 (3.8%)
Someone they personally knew being infected	
No	15 352 (99.5%)
Yes	76 (.5%)
*Cognitive factors*	
Perception that COVID-19 will be under control in the coming month	
Certainly not	70 (.5%)
Very small chance	180 (1.2%)
Small chance	986 (6.4%)
Large chance	2194 (14.2%)
Very large chance	5974 (38.7%)
Certainly yes	6024 (39.0%)
Perception that COVID-19 will pose long term physical harm to human	
Don’t know	5554 (36.0%)
No	962 (6.2%)
Yes	8912 (57.8%)
*Social factor*	
Social support score (mean, range 1 to 10)	Mean = 8.60, *SD* = 2.03

*COVID-19* Coronavirus disease 2019, *SD* Standard deviation, *M* Mean

### Mental health and preventive behaviours

Less than one-fifth (19.8%) reported always wearing a face mask when going out, a similar number (19.1%) reported washing hands with soap for 11 times or more per day. 43.6% and 28.2% scored higher than the cut-off for probable depression and probable anxiety respectively. The frequency of always wearing a face mask (15.1%) and washing hands with soaps for 11 times or more (13.6%) was lowest in Central China. The prevalence of depression (52.6%) and anxiety (35.6%) was highest in Northwest China (Table 2)*.*

**Table 2:** Mental health and personal preventive behaviors among pregnant women by region (*N* = 15 428)

	Total	Northern China	Eastern China	Southern China	Central China	Northwest China	Difference between groups
*Mental health*							
Depression (measured by PHQ-9)							*χ*^2^(16) = 76.91***
Minimal (0 to 4)	8705 (56.4%)	1481 (58.7%)	4350 (57.0%)	1662 (57.8%)	808 (52.3%)	404 (47.4%)	
Mild (5 to 9)	4402 (28.5%)	721 (28.6%)	2,160 (28.3%)	769 (26.8%)	494 (32.0%)	258 (30.3%)	
Moderate (10 to 14)	1549 (10.0%)	216 (8.6%)	764 (10.0%)	291 (10.1%)	160 (10.4%)	118 (13.8%)	
Moderately severe (15 to 29)	566 (3.7%)	79 (3.1%)	255 (3.3%)	112 (3.9%)	65 (4.2%)	54 (6.3%)	
Severe (20 to 27)	206 (1.3%)	28 (1.1%)	103 (1.3%)	39 (1.4%)	18 (1.2%)	18 (2.1%)	
Anxiety (measured by GAD-7)							*χ*^2^(12) = 62.47***
Minimal (0 to 4)	11 079 (71.8%)	1871 (74.1%)	5582 (73.1%)	2026 (70.5%)	1051 (68.0%)	549 (64.4%)	
Mild (5 to 9)	3258 (21.1%)	489 (19.4%)	1573 (20.6%)	610 (21.2%)	372 (24.1%)	213 (25.0%)	
Moderate (10 to 14)	734 (4.8%)	106 (4.2%)	325 (4.3%)	159 (5.5%)	85 (5.5%)	59 (6.9%)	
Severe (15 to 21)	357 (2.3%)	59 (2.3%)	152 (2.0%)	78 (2.7%)	37 (2.4%)	31 (3.6%)	
*Personal preventive behaviors*							
Frequency of wearing face mask when going out							*χ*^2^(12) = 76.33***
Never	5507 (35.7%)	837 (33.1%)	2607 (34.2%)	1134 (39.5%)	585 (37.9%)	344 (40.4%)	
Seldom	5416 (35.1%)	893 (35.4%)	2691 (35.3%)	970 (33.8%)	585 (37.9%)	277 (32.5%)	
Sometimes	1444 (9.4%)	279 (11.9%)	714 (9.4%)	233 (8.1%)	142 (9.2%)	76 (8.9%)	
Always	3061 (19.8%)	516 (20.4%)	1620 (21.2%)	536 (18.7%)	233 (15.1%)	155 (18.2%)	
Frequency of washing hands with soap per day							*χ*^2^(16) = 414.66***
0 to 2 times	1934 (12.5%)	138 (5.5%)	1002 (13.1%)	435 (15.1%)	302 (19.5%)	57 (6.7%)	
3 to 5 times	5917 (38.4%)	820 (32.5%)	2970 (38.9%)	1159 (40.3%)	648 (41.9%)	319 (37.4%)	
6 to 10 times	4634 (30.0%)	918 (36.4%)	2265 (29.7%)	750 (26.1%)	385 (24.9%)	316 (37.1%)	
11 to 15 times	1388 (9.0%)	314 (12.4%)	689 (9.0%)	204 (7.1%)	102 (6.6%)	79 (9.3%)	
More than 15 times	1555 (10.1%)	335 (13.3%)	706 (9.3%)	325 (11.3%)	108 (7.0%)	81 (9.5%)	

*PHQ-9* Patient Health Questionnaire-9, *GAD-7* General Anxiety Disorder-7

### Correlation between mental health and preventive behaviors

Results from correlation analyses showed a significant positive correlation between frequency of wearing face masks and mental health (*r* = 0.04 for depression and *r* = 0.03 for anxiety, *P* < 0.001). On the other hand, a significant negative correlation was found between frequency of washing hands with soap and mental health (*r* = −0.11 for depression and *r* = −0.08 for anxiety, *P* < 0.001) (Data not tabulated).

### Factors associated with mental health and preventive behaviours

*Probable depression.* The results of the simple logistic regressions showed that among the socio-demographic and pregnancy-related variables, older age (*OR* = 0.43–0.67), having child (ren) (*OR* = 0.63–0.76), and higher gestational age (*OR* = 0.71–0.72) were significant protective factors, while higher level of education (*OR* = 1.31–1.45) was a significant risk factor of probable depression.

Among the contextual, cognitive and social factors, being quarantined (*OR* = 1.40), having someone they knew being infected with COVID-19 (*OR* = 1.89), and perceiving that COVID-19 would pose long term physical harm to human (*OR* = 1.27) were significant risk factors, while perceiving that the COVID-19 would be under control in the coming month (*OR* = 0.63) and higher level of social support (*OR* = 0.86) were significant protective factors of probable depression. These variables remained significant after adjusting for significant socio-demographic and pregnancy-related variables as control variables. Place of residence being locked down also became statistically significant after adjusting for significant socio-demographic and pregnancy-related variables as control variables (a*OR* = 1.10) (Table 3).

**Table 3:** Logistic regression for mental health and personal preventive behaviours among pregnant women (*N* = 15 428)

	Probable depression (PHQ-9 > 5)		Probable anxiety (GAD-7 > 5)		Always wearing face mask		Washing hands with soap for > 11 times per day	
	*OR* (95% *CI*)	a*OR* (95% *CI*)	*OR* (95% *CI*)	a*OR* (95% *CI*)	*OR* (95% *CI*)	a*OR* (95% *CI*)	*OR* (95% *CI*)	a*OR* (95% *CI*)
*Socio-demographic characteristics*								
Age, years								
19 or below	1	-	1	-	1	-	1	-
20–25	0.74 (0.55–1.00)		0.57 (0.42–0.78)***		0.89 (0.61–1.30)		1.30 (0.98–1.91)	
26–30	0.67 (0.50–0.91)*		0.54 (0.40–0.73)***		0.99 (0.68–1.44)		1.57 (1.08–2.28)*	
31–35	0.62 (0.46–0.84)**		0.50 (0.37–0.68)***		0.98 (0.68–1.42)		1.90 (1.31–2.77)**	
36–40	0.46 (0.33–0.63)***		0.43 (0.31–0.59)***		1.00 (0.68–1.48)		2.31 (1.57–3.40)***	
41 or above	0.43 (0.29–0.64)***		0.42 (0.28–0.64)***		1.41 (0.89–2.26)		1.99 (1.26–3.16)**	
Education level								
Primary or below	1	-	1	-	1	-	1	-
Junior secondary	1.08 (0.83–1.39)		1.00 (0.76–1.32)		1.00 (0.72–1.39)		1.09 (0.82–1.44)	
Senior secondary	1.21 (0.94–1.57)		.96 (0.73–1.27)		.87 (0.62–1.21)		1.11 (0.84–1.47)	
Matriculation	1.31 (1.02–1.68)*		.96 (0.73–1.25)		1.15 (0.83–1.59)		1.13 (0.86–1.50)	
Undergraduate	1.38 (1.07–1.77)*		1.01 (0.77–1.33)		1.57 (1.14–2.17)**		1.11 (0.84–1.45)	
Postgraduate or above	1.45 (1.10–1.92)**		1.13 (0.84–1.53)		1.50 (1.06–2.13)*		1.15 (0.85–1.55)	
*Pregnancy-related characteristics*								
Parity								
Nulliparous	1	-	1	-	1	-	1	-
Primiparous	0.76 (0.71–0.81)***		0.84 (0.78–0.90)***		0.84 (0.77–0.91)***		1.23 (1.14–1.32)***	
Multiparous	0.63 (0.54–0.74)***		0.86 (0.73–1.02)		0.69 (0.56–0.84)***		1.19 (1.01–1.41)*	
Gestational age		-		-		-	-	-
1st trimester (12 weeks or below)	1		1		1		1	
2nd trimester (13 to 26 weeks)	0.72 (0.64–0.81)***		0.84 (0.74–0.95)**		0.86 (0.76–0.99)*		1.13 (0.99–1.29)	
3rd trimester (27 weeks or above)	0.71 (0.64–0.80)***		0.94 (0.83–1.06)		0.70 (0.61–0.80)***		1.33 (1.17–1.51)***	
Pregnancy-related complications								
No	1	-	1	-	1	-	1	-
Yes	0.99 (0.99–1.11)		1.28 (1.13–1.44)***		0.94 (0.82–1.08)		1.22 (1.09–1.38)**	
*Contextual factor*								
Place of residence being locked down								
No	1	1	1	1	1	1	1	1
Yes	1.04 (0.98–1.11)	1.10 (1.03–1.18)**	1.09 (1.02–1.18)*	1.11 (1.03–1.20)**	0.81 (0.75–0.88)***	0.91 (0.84–1.00)	0.99 (0.90–1.04)	0.99 (0.92–1.07)
Being quarantined								
No	1	1	1	1	1	1	1	1
Yes	1.40 (1.19–1.65)***	1.42 (1.20–1.68)***	1.56 (1.31–1.85)***	1.57 (1.32–1.86)***	1.03 (0.83–1.26)	1.05 (0.85–1.29)	0.97 (0.81–1.17)	0.99 (0.82–1.19)
Someone personally knew being infected								
No	1	1	1	1	1	1	1	1
Yes	1.89 (1.19–2.98)**	1.80 (1.14–2.85)**	1.96 (1.25–3.09)**	1.92 (1.22–3.03)**	0.76 (0.41–1.40)	0.69 (0.37–1.28)	0.76 (0.44–1.29)	0.75 (0.44–1.28)
*Cognitive factors*								
COVID-19 will be under control in the coming month								
Certainly not / very small / small chance	1	1	1	1	1	1	1	1
High / very high chance / certainly yes	0.63 (0.56–0.70)***	0.63 (0.56–0.71)***	0.59 (0.52–0.67)***	0.59 (0.53–0.67)***	0.84 (0.73–0.97)*	0.85 (0.74–0.98)*	1.03 (0.91–1.18)	1.04 (0.91–1.18)
COVID-19 will pose long term physical harm to human								
Don’t know /no	1	1	1	1	1	1	1	1
Yes	1.27 (1.19–1.35)***	1.25 (1.17–1.33)***	1.30 (1.21–1.39)***	1.28 (1.20–1.38)***	1.08 (1.00–1.17)*	1.06 (0.97–1.14)	0.98 (0.91–1.06)	0.98 (0.92–1.06)
*Social factor*								
Social support score (mean, range 1 to 10)	0.86 (0.85–0.87)***	0.86 (0.85–0.88)***	0.86 (0.85–0.88)***	0.87 (0.85–0.88)***	1.01 (0.99–1.03)	1.00 (0.99–1.03)	1.06 (1.04–1.08)***	1.06 (1.04–1.08)***

*OR* Odds ratio derived from univariate logistic regression, a*OR* Odds ratio derived from univariate logistic regression, adjusting for socio-demographic and pregnancy-related characteristics that were significant in the univariate logistic regression, *CI* Confidential interval

^*^P < 0.05, **P < 0.01, ***P < 0.001

*Probable anxiety.* The results of the simple logistic regressions showed that among the socio-demographic and pregnancy-related variables, older age (*OR* = 0.42–0.57), being primiparous (*OR* = 0.84), and being at the 2nd trimester (*OR* = 0.84) were significant protective factors, while having pregnancy-related complications (*OR* = 1.28) was a significant risk factors of probable anxiety.

Among the contextual, cognitive and social factors, place of residence being locked down (*OR* = 1.09), being quarantined (*OR* = 1.56), having someone they knew being infected with COVID-19 (*OR* = 1.96), and perceiving that COVID-19 would pose long term physical harm to human (*OR* = 1.30) were significant risk factors, while perceiving that the COVID-19 would be under control in the coming month (*OR* = 0.59) and higher level of social support (*OR* = 0.86)were significant protective factors of probable anxiety. These variables remained significant after adjusting for significant socio-demographic and pregnancy-related variables as control variables (Table 3).

*Always wearing a face mask when going out.* The results of the simple logistic regressions showed that among the socio-demographic and pregnancy-related variables, higher level of education (*OR* = 1.50–1.57) was a significant protective factor, while having child(ren) (*OR* = 0.69–0.84) and higher gestational age (*OR* = 0.70–0.86) were significant risk factors of always wearing a face mask.

Among the contextual, cognitive and social factors, place of residence being locked down (*OR* = 0.81) and perceiving that the COVID-19 would be under control in the coming month (*OR* = 0.84) were significant risk factors, while perceiving that the COVID-19 would pose long term physical harm to human (*OR* = 1.08) was a significant protective factor of always wearing face mask when going out. All variables except the perception that COVID-19 would pose long term physical harm to human remained significant after adjusting for significant socio-demographic and pregnancy-related variables as control variables (Table 3).

*Washing hands with soap for* > *11 times per day.* The results of the simple logistic regressions showed that among the socio-demographic and pregnancy-related variables, older age (*OR* = 1.57–2.31), having child(ren) (*OR* = 1.19–1.23), being in the 3rd trimester (*OR* = 1.33) and having pregnancy-related complications (*OR* = 1.22) were significant protective factors of washing hands with soap for > 11 times per day.

Among the contextual, cognitive and social factors, only social support emerged as a protective factor of washing hands with soap for > 11 times per day (*OR* = 1.06). It remained significant after adjusting for significant socio-demographic and pregnancy-related variables as control variables (Table 3).

## Discussion

The current study was conducted at the early phase of the COVID-19 period before worldwide recommendation of the use of face masks in the general population was made. Findings reported that only one-fifth (19.8%) of the participants reported always wearing face mask when going out, and a similar number (19.1%) reported washing hands with soap for more than 10 times per day. The prevalence was lower then that documented in other respiratory epidemics, such as SARS and H1N1 [20, 21]. It was also significantly lower than those reported in a study conducted in Wuhan and Shanghai during the early phase of the COVID-19 outbreak [10]. It is also important to note that 28.2% and 43.6% of the current sample scored above the cut-off for anxiety and depression respectively. The prevalence was higher than those reported in other studies among pregnant women [12, 22]. Findings suggest that an epidemic outbreak may increase the risk for depression and anxiety of pregnant women. Regions differences in mental health and preventive behaviours were also observed. The frequency of always wearing a face mask and washing hands with soaps for 11 times or more was lowest in Central China, and the prevalence of depression and anxiety was highest in Northwest China. Such results were not surprising as these regions have been one of the most significantly affected areas or geographically close to such areas in the early phase of COVID-19. Health care professionals should pay special attention to the mental health and personal preventive behaviour of pregnant women during an epidemic outbreak.

A large number of pregnant women (91.9%) perceived that the COVID-19 would be under control in the coming month, and slightly more than half (57.8%) agreed that COVID-19 would pose long term physical harm to human. The figures were very different from that documented in other epidemics, such as the SARS and H1N1 that large number of individuals believed that there was no effective treatment available to control for the disease, and that the disease would pose long term body damage [23, 24]. As China has confronted several pandemic outbreaks in the past decades, it might be possible that the current sample has become rational in responding to COVID-19. Also, the government has adopted very radical measures (e.g. city lockdown) to prevent the spread of the disease when the survey was conducted, it might have increased the participants’ belief that the disease will be under control in a short period.

The present study shows that among the socio-demographic variables, older age was associated with lower risk of depression and anxiety. Such findings were in contrast with previous studies older age was a risk factor for depression among pregnant women [22]. Older women might have the experiences of coping with prior pandemics such as the SARS and H1N1, they might therefore be more likely to show higher resilience when facing the challenge of another epidemic. Older age was also found to be associated with more frequent handwashing behaviours; a finding that was consistent with the extant literature [25, 26].

Furthermore, women with higher level of education were more likely to have probable depression and report always wearing mask when going out. Such findings were again, in contrast with previous studies that revealed negative association between depression and education level among pregnant women [22]. Pregnant women with a higher level of education might be more active in seeking for information about COVID-19 [27], they might therefore be more well-informed with the risk and harm of COVID-19, and subsequently more likely to report feelings of worry, anxiety and depression but also higher tendency in engaging in personal preventive behaviour. Women with child(ren) and higher gestational age were also more likely to report better mental health, higher frequency handwashing behaviour but lower frequency of always wearing face mask. More studies are needed to delineate the contradictory findings found in personal preventive behaviours. All in all, findings highlight the important role of socio-demographic and pregnancy-related factors on health risk among pregnant women during an epidemic outbreak.

Among the contextual factors, place of residence being locked down, being quarantined and personally knowing someone being infected with COVID-19 were associated with worse mental health. It is conceivable that those who were living in city being locked down or being quarantined might indicate a higher risk of being infected with COVID-19. Knowing someone who were being infected might also increase their perceived susceptibility of the infection, which adversely affect their mental health [28]. Interestingly, these factors had no significant association with personal preventive behaviours, suggesting that such measures might have increased the public’s fear about being infected but had no effect on their behavioural coping responses to the disease.

There is extensive evidence that disease-related perceptions are crucial in driving behavioural and emotional response to a disease [29, 30]. In the present study, the belief that disease would be under control was associated with better mental health but also lower frequency of wearing a face mask. Those who believe that the disease will be under control tended to be more optimistic about the epidemic, therefore less likely to show negative emotional responses and precautions. On the other hand, the belief that the disease will pose long term physical harm was associated with worse mental health. Results were consistent with previous studies in respiratory epidemics that perceiving the disease would cause permanent bodily damages or highly fatal were significantly associated with worse mental health [31].

The positive influence of social support on mental health and health behaviours has been widely demonstrated in various populations, including the pregnant women [12, 13]. Consistent with the literature [25], the present study showed that social support was associated with better mental health and higher frequency of washing hands with soap during an epidemic outbreak. Individuals with higher level of social support during the epidemic outbreak might have received more cognitive, emotional and tangible resources in meeting the adversities, which prevented risk of mental health problems. In addition, individuals’ family, friends, or significant others can act as positive models or social control for health behaviours, or provide encouragement for engaging in personal preventive behaviours. Findings of the present study provided further evidence on the important role of social support on health during an epidemic outbreak.

The present study has identified some groups of pregnant women who would be particularly vulnerable to poor mental health and lower levels of preventive behaviours. Assessing the socio-demographic and background factors would therefore be particularly important for early identification and management of health risk among pregnant women. The present study also demonstrated that locking down a city and quarantining those at high risk of infection might have significant detrimental impact on individual mental health. Policy makers should be aware of how the public health policy would have an impact on mental health of the population. The significant association between disease perceptions and preventive behaviours highlighted the importance of educating the pregnant women about the risk and negative consequences of COVID-19. On the other hand, it is also important that such education materials should not create unrealistic or exaggerated fears about the condition as they may increase risk for mental health problems. Finally, the protective role of social support on mental health and preventive behaviors suggested that promoting social support would be essential in promoting mental health and personal preventive behaviors among pregnant women during an epidemic outbreak. Interventions to promote social support could involve women and their family members in family planning, provide women and their family members with stress management training, improve skills in coping with the epidemic outbreak and preparation for the pregnancy, and recommend strategies for maintaining supportive relationships during the pregnancy.

There were several limitations that should be noted. First, the present study was cross-sectional in nature so causality could not be assumed. Second, the number of responses varied substantially between provinces so the samples might not be representative. Generalizability of the findings might have been limited. Third, due to the lack of validated scales on perceptions of infectious diseases and other contextual factors, self-developed items were used. Fourth, single item was used due to time constraint on an online survey, the reliability of the measure was therefore limited. Finally, data were self-reported so the prevalence of mental health and personal preventive behaviours might have been over-estimated.

## Conclusions

The present study revealed that the prevalence of probable depression and anxiety was high while the prevalence of wearing face mask and hand washing was low among pregnant women during the COVID-19 outbreak. Their mental health and personal preventive behaviours could be explained by a range of socio-demographic, pregnancy-related, contextual, cognitive and social factors. It would be important to assess the socio-demographic and pregnancy-related factors to identify women who might be at risk for poor mental health and preventive behaviours during a pandemic. Interventions to mitigate their mental health problems and to promote preventive behaviours are highly warranted and they should seek to provide education on risk and consequences of COVID-19, and increase social support.

## Supplementary Information

**Additional file 1.** Measures used in the present study.

## Data Availability

The data supporting the findings of the present study is unavailable to share, as it is the requirement of the ethics committee that only the research team can get access to the data.

## Abbreviations

COVID-19: Coronavirus disease 2019
OR: Odd ratio
aOR: Adjusted odd ratio
SARS: Severe acute respiratory syndrome
MERS: Middle East respiratory syndrome
H1N1: Influenza A virus subtype
WHO: World Health Organisation
QR: Quick response
PHQ-9: Patient Health Questionnaire-9
GAD-7: General Anxiety Disorder-7
