# Supplementary material for: Mental health and preventive behaviour of pregnant women in China during the early phase of the COVID-19 period
Source: Infect Dis Poverty. 2021 Mar 24;10:37. doi: 10.1186/s40249-021-00825-4 (PMC7988630; doi:10.1186/s40249-021-00825-4)
Supplement: Supplementary file 1 — Additional file 1. Measures used in the present study. [file 40249_2021_825_MOESM1_ESM.docx]

Additional file

*Measures*

Socio-demographic characteristics including age, education level, and place of residence, and pregnancy-related characteristics including parity, gestational age, and whether they had any pregnancy-related complications were collected. Participants were also asked to report whether their place of residence was locked down, whether they have been quarantined, and whether someone they personally knew were infected with COVID-19.

Cognitive factors. Two items related to perceptions of COVID-19 were asked. Participants were asked to rate the likelihood that the COVID-19 would be under control in the coming month on a 6-point Likert Scale (1 = certainly not to 6 = certainly yes). They were also asked to rate if the COVID-19 would pose long term physical harm to human (don’t know, no, yes). Similar item was used in a number of published papers that assessed perceptions related to other diseases (18).

Social factor. Participants were asked to rate the level of social support they have obtained during the COVID-19 outbreak period on a 10-point Likert Scale (1 = very low to 10 = very high). Similar item was used in a number of published papers (19).

Personal preventive behaviors. Participants were asked to report their frequency of wearing a face mask when going out on a 4-point Likert Scale (1 = never to 4 = always). A cut-off of always wearing mask was set in the present study. They were also asked to report their frequency of washing hands with soap everyday on 5-point Likert Scale (1 = 0-2 times, 2 = 3-5 times, 3 = 6-10 times, 4 = 11-15 times, 5 = more than 15 times). A cut-off of more than 11 times per day was set in the present study; similar cut-off has been used in a number of published papers (20, 21).

Mental health. Depression was measured by the 9-item Chinese version of Patient Health Questionnaire-9 (PHQ-9) (22). Respondents are asked if each representing symptom of major depressive disorder has ever occurred to them over the past 2 weeks, with responses ranging from 0 ‘not at all’ to 3 ‘nearly every day’. The total score ranges from 0 to 27, and a score of 0 to 4, 5 to 9, 10 to 14, 15 to 19, and 20 to 27 represents minimal, mild, moderate, moderately severe, and severe depression. Anxiety was assessed by the 7-item Chinese version of General Anxiety Disorder scale (GAD-7) (23). These 7-item inquires about anxiety-related symptoms in the past 2 weeks. The response options of GAD-7 are identical to those of PHQ-9. The GAD-7 total score ranges from 0 to 21, with higher scores representing more severe anxiety. A score of 0 to 4, 50 to 9, 10 to 14, and 15 to 21 represents minimal, mild, moderate, and severe anxiety.
